# Correlations in background activity control persistent state stability and allow execution of working memory tasks

**DOI:** 10.3389/fncom.2013.00139

**Published:** 2013-10-21

**Authors:** Mario Dipoppa, Boris S. Gutkin

**Affiliations:** ^1^Departement d'Etudes Cognitives, Ecole Normale Superieure, Group for Neural Theory, Laboratoire des Neurosciences Cognitives INSERM U960Paris, France; ^2^Ecole Doctorale Cerveau Cognition Comportement, Université Pierre et Marie CurieParis, France; ^3^Centre national de la recherche scientifiqueParis, France

**Keywords:** correlations, background activity, working memory, spiking neural network, persistent activity

## Abstract

Working memory (WM) requires selective information gating, active information maintenance, and rapid active updating. Hence performing a WM task needs rapid and controlled transitions between neural persistent activity and the resting state. We propose that changes in correlations in neural activity provides a mechanism for the required WM operations. As a proof of principle, we implement sustained activity and WM in recurrently coupled spiking networks with neurons receiving excitatory random background activity where background correlations are induced by a common noise source. We first characterize how the level of background correlations controls the stability of the persistent state. With sufficiently high correlations, the sustained state becomes practically unstable, so it cannot be initiated by a transient stimulus. We exploit this in WM models implementing the delay match to sample task by modulating flexibly in time the correlation level at different phases of the task. The modulation sets the network in different working regimes: more prompt to gate in a signal or clear the memory. We examine how the correlations affect the ability of the network to perform the task when distractors are present. We show that in a winner-take-all version of the model, where two populations cross-inhibit, correlations make the distractor blocking robust. In a version of the mode where no cross inhibition is present, we show that appropriate modulation of correlation levels is sufficient to also block the distractor access while leaving the relevant memory trace in tact. The findings presented in this manuscript can form the basis for a new paradigm about how correlations are flexibly controlled by the cortical circuits to execute WM operations.

## Introduction

Working memory (WM), defined as short term storage of information that is actively used on-line to carry out actions and decisions and drive learning, is one of the key processes that underpins our cognitive abilities. WM is characterized by an information bottleneck with resources restricting its “on-line” capacity to a relatively limited number of items at high levels of performance (Miller, 1956; Luck and Vogel, 1997; Cowan, 2001; Vogel et al., 2001) and a rapid decrease in performance with item number due to limited resource allocation (Wilken and Ma, 2004; Bays and Husain, 2008; van den Berg et al., 2012) as suggested by the recent experiments. Furthermore by its very nature, WM is characterized by the need to operate on the stored information rapidly. Such limitations and rapid operations of WM create the need for selective gating and rapid updating as well as active information maintenance to enable its immediate use (Frank et al., 2001). One of the central unresolved issues is how the multiple requirements for WM are carried out by the brain circuits: whether the maintenance, read-in, gating, and read-out are implemented by separated systems (e.g., as suggested by Baddeley, 2003) or by operations within the same neural circuit (e.g., as recently put forward by Machens et al., 2005).

Electrophysiological data from primate performing delayed-response tasks show that persistent neuronal activity in prefrontal cortex (PFC) underlies the maintenance of WM: during the delay period between the stimulus presentation and the read-out, neurons selective to the memorized stimulus fire spikes at an elevated rate with respect to the resting state (Fuster and Alexander, 1971; Fuster and Jervey, 1981; Funahashi et al., 1989; Miller et al., 1996; Romo et al., 1999).

**Figure 1:**
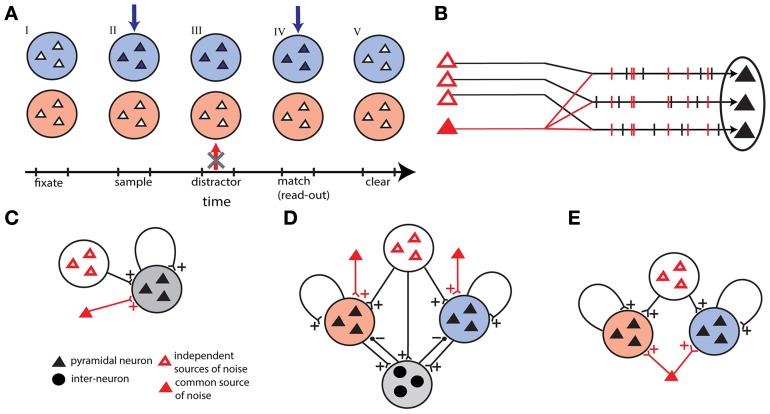
**Outline of the models. (A)** Time sequence of the delay match-to-sample task for the working memory network. Active neurons are represented in full colors. Successively: (I) both populations are in a quiescent state, (II) sample stimulus (blue arrow) activates blue population, (III) the network prevent a distracting stimulus (red arrow) to activate the red population, (IV) match stimulus allows the read-out of the encoded memory in the blue population, and (V) persistent activity is erased in the blue population. **(B)** Correlations in external background activity generated by a common source of noise, in addition to independent sources of noise. **(C)** Single unit network receiving shared and independent sources of noise. **(D)** Winner-take-all network with two competing excitatory populations coupled through one inhibitory population. In addition to independent sources the excitatory populations receive background activity by two different common noise sources. **(E)** Two-unit network with two excitatory populations receiving shared noise.

In order to highlight the unique requirements of the WM as a neural process let us focus on the DMS task with distractors as a prototypical example (Miller et al., 1996). In this task the subject must remember the identity of an item briefly shown (the sample) and respond correctly only when the item is shown again (match) all the while ignoring other items flashed (distractors). To execute correctly this task, the neural circuitry needs to perform three operations (Figure 1A): first, encode and maintain in memory the sensory stimulus during the delay period; second, robustly maintain the memory face to distractors presentation; third, erase the memory trace at task completion to make the store available again, given the limited WM capacity. These operations are translated in terms of neural activity as follows: item-related activity is turned on rapidly and selectively by the sample-stimulus, is protected from distractors during the delay period, and is rapidly turned off on response by the match.

A number of spiking network models have been conceived to describe the neural substrate for WM where persistent activity is maintained by recurrent connections that allow for co-existing attractor memory states and a ground non-memory state (Amit and Brunel, 1997; Compte et al., 2000; Brunel and Wang, 2001; Gutkin et al., 2001; Laing and Chow, 2001; Machens et al., 2005; Miller and Wang, 2006; Ardid et al., 2010). In some of these models, protection from distractors and memory clearance are performed through the recruitment of inhibition (Compte et al., 2000; Brunel and Wang, 2001; Machens et al., 2005). As an alternative to the erasing-by-inhibition paradigm, it has been shown, in a spatial WM model, that a transient excitatory stimulus matching the memory trace “location” on the network extinguishes the persistent state by transiently synchronizing the spike-times of the neurons (Gutkin et al., 2001; Laing and Chow, 2001). This work, along with Machens et al. (2005) showed how the read-out and clear-out can be merged into a single operation. However, in these alternative frameworks, protection from distractors, or selective gating, was not addressed. Here we propose that the gating is obtained by flexibly controlling the spike-time structure of the WM network activity. In support of this idea, it has been shown that spike-time synchronization is modulated in association with cognitive processing (Abeles et al., 1993; Riehle et al., 1997; Funahashi and Inoue, 2000) and in particular in WM (Sakurai and Takahashi, 2006; Pipa and Munk, 2011).

Critically, WM trace appears in the context of on-going background activity. While background activity is not related to task parameters, this is not without structure. Correlations have been found broadly in spontaneous neural activity in the cortex (Tsodyks et al., 1999). In particular, it has been shown that nearby neurons receive common inputs from afferent neurons making their voltages correlated (Lampl et al., 1999). Effects of correlations have been widely studied for their effect on population code (Salinas and Sejnowski, 2001), to measure network connectivity (Aertsen et al., 1989; Cocco et al., 2009), on neural dynamics for coupled neurons (Ly and Ermentrout, 2009), and for multiple independent neurons (Galán et al., 2006; Moreno-Bote et al., 2008).

In computational models of WM the background activity has been largely seen as problematic for memory maintenance. For example one of the more sensitive technical issues addressed by several computational proposals is how to stabilize the WM trace in face of random background activity (Compte et al., 2000). The benefits of external input correlations on persistent activity in recurrent networks have only recently started to be addressed theoretically (Buice et al., 2010; Polk et al., 2012). For the specific case of line-attractor networks (modeling parametric WM) Polk et al. (2012) showed in a detailed analysis how properly tuned input noise correlations can promote stability of the persistent firing rate. This was further noted in Lim and Goldman (2012) who also showed that the correlation structure of background noise can suggest the optimal architecture of neural networks for short term memory performance.

Finally, in this article we examined the influence of input correlations on recurrent spiking networks, finding that the correlation level in fact may destabilize the persistent activity state, rendering it a slow transient state. Buice et al. (2010) used a path integral approach to integrate the effects of correlations and synchronization into a rate model of recurrent networks and examined the stability of the persistent state. For a bistable firing-rate network they noted that transient increases in input correlations (synchronizing noise input) can lead to a turn-off of the persistent activity. This approach may in fact provide an analytical framework of the observations we make in the present manuscript for recurrent spiking networks and the correlation-based control of the persistent state lifetime. In this manuscript we also go beyond noting that input correlations defined the lifetime of persistent activity; we show that input correlations can effectively control the access to WM by disallowing transient stimuli to initiate persistent activity. The functional consequences of these two effects are the central topic of this work.

To demonstrate that, by controlling the correlation-driven synchronization of the background activity it is possible to control the lifetime of the persistent state and to manipulate selectively the transitions in sustained activity and consequently to perform the required operations of the WM task, we first consider a minimal recurrent network. In this recurrent network the neurons receive an excitatory random background noise, and background correlations are induced by a common noise source. Then we implement a discrete item WM model where the modulation of the background correlation level sets the network into different regimes allowing for loading of memory, protection from distractors and memory persistence. In addition we show the possibility to merge the read-out and the clearance in a single operation since the presentation of the match stimulus can directly quench the persistent activity.

## Materials and methods

### Neural models

In this work we study recurrent spiking networks that show bistability between a ground state and an active persistent spiking state. Our goal is to construct and analyze a minimal network capable of showing the required bistability. Hence we consider networks of recurrently connected excitatory pyramidal neurons. The elements of the network are represented by non-linear “point” neurons that are sparsely connected by instantaneous excitatory recurrent synapses. The dynamics of a neuron's membrane potential *v* is described by the Quadratic Integrate and Fire (QIF) equation, which represents the normal form of type 1 spike generating dynamics (Ermentrout, 1996):

(1) $$\tau \frac{dv}{dt}=v^{2}-b^{2}+I_{\text{syn}}(t)$$

(2) $$v(t)=V_{t}\to V_{r}$$

where τ represents the membrane time constant, −*b* is the resting potential, *I*(*t*) the input current, *V*_*t*_ a spike threshold, and *V*_*r*_ the reset membrane potential. The voltage of the neuron is scaled such that *v* is a non-dimensional variable. When the membrane potential neuron attains the threshold value *v* = *V*_*t*_, a spike is emitted and a post-synaptic current (PSC) is transmitted to an output neuron. We set the parameters as follows: *V*_*r*_ = −20, *V*_*t*_ = 20, *b* = 1 and τ = 20 ms.

The input current to a given cell in the network is decomposed into three different components:

(3) $$I_{\text{syn}}(t)=I_{r}(t)+I_{s}(t)+I_{\text{ba}}(t)$$

where *I*_*r*_(*t*) represents the recurrent input due to other neurons in the network, *I*_*s*_(*t*) represents the input from external stimuli directed to the network, and *I*_ba_(*t*) represents a non-specific background activity. Each of the three currents corresponds to a sum of PSCs originating from synaptic inputs generated by the presynaptic neurons at times *t*_*n*_. The PSCs are modeled with delta pulses:

(4) $$I(t)=\sum_{a}\sum_{\left\{t_{n}\right\}}J_{a}\tau \delta (t-t_{n})$$

where *J*_*a*_ represents the synaptic strength for a given connection and could be positive (corresponding to an AMPA synapse) or negative (corresponding to a GABA synapse).

### Background activity and correlations measures

Ample data shows that cortical neurons receive a large amount of non-specific cortical and subcortical inputs whose structure is not directly related to the specific task and stimulus [e.g., see Shadlen and Newsome (1994) and summary of data in Amit and Brunel (1997)]. We refer to this type of input as an external background activity. It is taken to be composed of sequences of excitatory PSCs of synaptic strength *J*_0_ and with the synaptic times generated by a Poisson process. The synaptic currents are depolarizing in accordance with the notion that cortical neurons receive inputs from long-range excitatory glutamatergic projections.

In our model, this background activity can be either unstructured (uncorrelated) or structured (correlated). The correlation level, between two spike trains *S*_*i*_(*t*) and *S*_*j*_(*t*) is given by:

(5) $$\lambda_{ij}=\frac{1}{\langle S_{i}(t)\rangle }\int {\text{CCVF}}_{ij}(s)ds$$

where CCVF corresponds to the cross-covariance function (Brette, 2009). This function is normalized to zero if *S*_*i*_(*t*) and *S*_*j*_(*t*) are generated by independent Poisson processes.

We consider two ways for constructing the background activity:

#### Uncorrelated background activity

All *N* neurons receive spike trains generated by *N* independent channels with rate ν_0_. This leads to CCVF(*s*) = 0 and thus the correlation level is λ_*ij*_ = 0.

#### Correlations induced by a common source of noise (Figure 1B)

All the *N* neurons receive inputs both from independent channels, with frequency (1 − λ) ν_0_, and from a common channel, with frequency λ ν_0_ and 0 ≤ λ ≤ 1. Each channel generates a spike train with Poisson statistics. The average background input rate is ν_0_ for each neuron. The cross-covariance function is then CCVF(*s*) = λ 〈*S*_*i*_(*t*)〉δ(*s*) and the correlation level is λ_*ij*_ = λ. This gives purely spatial correlations.

We measure the correlation level of the synaptic input among cells in the network with the mean Pearson correlation coefficient. We first compute a running mean (averaged over a time window of 5 ms) of the synaptic input *I*^*i*^_*a*_(*t*) for each cell during a certain interval of time. Then we compute the Pearson correlation between the synaptic input of two cells:

(6) $$\rho_{ij}=\frac{\text{cov}(I_{a}^{i},I_{a}^{j})}{\sigma (I_{a}^{i})\sigma (I_{a}^{j})}$$

Finally we compute the average over all the cell pairs of the network ρ = [2/*N*(*N* − 1)] ∑ *N*_*i* = 1_∑^*N*^_*j* = *i* + 1_ρ_*ij*_. In particular, in Figure 4, we performed this measure for the recurrent input *a* = *r* and background input *a* = *ba*.

### Functional network structures implementing WM tasks

In this work we study three different networks. We start out by studying a homogeneous network of recurrently coupled excitatory neurons. This network can be also thought of as a encoding a single item of WM: a “single-unit network”. The second model consists of two homogeneous excitatory networks coupled together through a population of inhibitory neurons: a “winner-take-all network” of two discrete competing short-term memory items. The third model is made up of two recurrent excitatory populations without mutual connections: a “two-unit network”.

#### Single-unit network

A homogeneous network with *N* = 100 identical sparsely coupled neurons is represented in Figure 1C. Each neuron in the network receives synaptic inputs from *cN* other excitatory neurons, where *c* = 0.2 is the probability of connection, and *J* = 0.26 is the recurrent synaptic strength [described in Equation (4)]. Neurons receive excitatory inputs also from external background activity, with synaptic strength *J*_0_ = 0.151 and firing rate ν_0_ = 106 Hz. Neurons also receive an excitatory input from external sensory stimuli with synaptic strength *J*_1_ = 1.5 and firing rate ν_1_ = 56 Hz for a duration of 50 ms, as will be described hereafter. Parameters of the network are chosen such that the network sustains a quiescent state, with low firing rate (*f* < 5 Hz), and a persistent state, with high firing rate (≈20 Hz).

#### Winner-take-all network

The second model is a reduced version of the network proposed by Amit and Brunel (1997) (Figure 1D). The network is composed of two excitatory populations and one inhibitory neural population. Each of the two excitatory populations has *N*_*E*_ = 40 neurons, and the third population is made up of *N*_*I*_ = 20 inhibitory neurons. An excitatory neuron receives synaptic inputs from *c*_EE_*N*_*E*_ (*c*_EE_ = 0.45) neurons of the same population, with synaptic strength *J*_EE_ = 0.3, and from *c*_EI_*N*_*I*_ (*c*_EI_ = 0.35) inhibitory neurons with synaptic strength *J*_EI_ = −0.25. An inhibitory neuron receives synaptic inputs from *c*_IE_*N*_*E*_ (*c*_IE_ = 0.34) excitatory neurons with synaptic strength *J*_IE_ = 0.05 from each excitatory population. Other parameters of the network are: *J*_0_ = 0.4, *J*_1_ = 1.5, ν_0_ = 60 Hz, and ν_1_ = 17 Hz. In addition we augment the mutual inhibition network with the added feature to control the amount of correlated noise in each excitatory population. More precisely each excitatory population receives background activity by common noise sources in addition to independent sources. In such a way the correlation level λ is regulated independently in each excitatory population.

#### Two-unit network

We devised a third version of our network models that is made of two excitatory independent populations, each one making recurrent connections with itself. Both networks share a common excitatory noise source projecting simultaneously to all excitatory neurons in addition to the independent uncorrelated background noise (Figure 1E). Since the common noise source is shared between the two populations the correlation level λ varies equally in the two excitatory populations. The parameters of each excitatory population are those given for the single-unit network, with the only difference that we used here a larger network (*N* = 1000) and we scaled accordingly the recurrent synaptic strength (*J* = 0.026).

### Delayed match-to-sample task

We study the spike-timing based mechanisms able to implement the DMS task (Figure 1A). The sequence of operations and the neural dynamics aim to reproduce the experimental results of Miller et al. (1996). For illustrative purposes, the discrete items can be viewed as corresponding to colors. The activation of one excitatory population encodes color blue, that we define population *B*, while the other encodes color red, that we define population *R*. If the populations are both in a quiescent state, the state of the network represents the absence of color information. For simplicity we represent the spontaneous state as the quiescent state (average firing rate ≈0 Hz).

During the task, the animal has to maintain a memory of an item (a color) during the delay period. In terms of neural activity, the corresponding excitatory population should be activated and maintained in a persistent sate. Additionally the model should protect the memory from the presentation of a distractor stimulus. At task completion after the decision, the system should erase rapidly the memory, i.e., the persistent activity should be deactivated to its quiescent state.

To establish that a network performs a WM task correctly we require it to perform all the operations of the task. The first operation, *load*, corresponds to loading the memory by the sample signal, and corresponds to *B* that is activated in a persistent state while *R* is in a quiescent state. The second operation, *protect*, corresponds to the maintenance of the blue item memory in the face of the distractor presentation. In terms of activity it corresponds to *B* maintained in the persistent state and *R* that is not activated to the persistent state even when the red stimulus is presented during the delay period. In networks (Figure 1E) where population *B* and population *R* are not connected, the operation *protect* can be separated in two independent sub-operations: *maintain* (maintain item memory in population *B*) and *block* (prevent activation of population *R*). The third operation, *clear* corresponds to the clearance of the memory encoded in the network. This is equivalent to the erasing of the persistent activity in the network. Note that in this work we do not focus explicitly on the read-out mechanism following the presentation of the match stimulus.

In particular in the winner-take-all network (resp. the two-unit network), operation *load* is executed with success if the sample stimulus activates population *B*. This is measured before distractor presentation during 350–450 ms (resp. 350–450 ms): ν_*B*_ > 5 Hz and ν_*R*_ < 5 Hz, where ν_*B*_ < 5 and ν_*R*_ denote the average population firing rates of populations blue and red, respectively. Operation *protect* is executed with success if population *B* maintains the persistent state and population *R* is not activated. This is measured before match presentation during 750–850 ms (resp. 700–800): ν_*B*_ > 5 Hz and ν_*R*_ < 5 Hz. Operation *clear* is executed with success if population *B* is deactivated at task completion. This is measured during an interval after match presentation, during 1150–1250 ms (resp. 1050–1150 ms): ν_*B*_ < 5 Hz and ν_*R*_ < 5 Hz.

### Numerical analysis

All the numerical results are obtained by algorithms run in Python. The differential equations are integrated with Euler steps of *dt* = 0.1 ms. The mean population firing rate *f* is computed over population average in 10 ms.

Data points for networks and associated error bars are computed by averaging over simulated individual network realizations. We generated random connectivity matrices such that every neuron receives the same number of input connections. For a fixed network connectivity matrix, we computed the average over 100 realizations of background activity and stimuli for each of 30 random realizations of the network connectivity matrix when not otherwise stated.

## Results

### Effects of correlations on persistent activity state in the single-unit network: erasing and blocking the memory trace

We examine how correlations in the background activity control selective persistent activity in WM networks. Hence we start out by analyzing how background correlations affect the transitions between the quiescent and self-sustained states in our network model.

**Figure 2:**
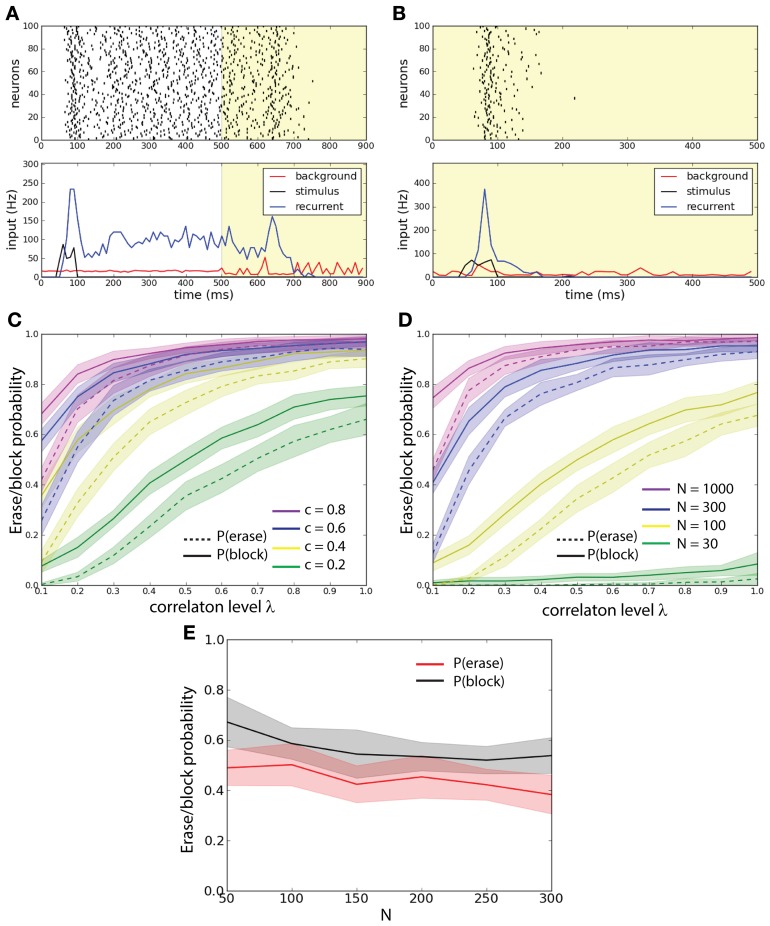
**External background correlations destabilize the persistent state in a single-unit network. (A)** Erasing persistent state with correlations. Examples of firing rate (top), and average synaptic input for one trial. λ = 0 until 500 ms and λ = 0.8 after 500 ms (yellow shaded areas). The population is activated by a stimulus during 50–100 ms. Correlations in background activity erase the persistent state. **(B)** Correlations gate the activation of the persistent state. λ = 0.8 all the time (yellow shaded areas). The network receives an excitatory stimulus during 50–100 ms. The stimulus fails to activate the persistent state in presence of background correlations. **(C)** Erasing probability *P*_*e*_ (continuous lines) and blocking probability *P*_*b*_ (dashed lines) as function of *c* and λ. Synaptic strength is scaled such that *cJN* = 0.52. Both *P*_*e*_ and *P*_*b*_ increase with increasing λ and *c*. **(D)** *P*_*e*_ (continuous lines) and *P*_*b*_ (dashed lines) as function of *N* and λ. Synaptic strength is scaled such that *cJN* = 0.52. Both *P*_*e*_ and *P*_*b*_ increase with increasing λ and *N*. **(E)** *P*_*e*_ and *P*_*b*_ as function of *N* with fixed λ = 0.6 and *J* = 0.26 and with *c* scaled such that *cN* = *const* = 20. *P*_*e*_ and *P*_*b*_ remain approximately constant.

Correlations in background activity are generated by the addition of a common noise source to independent stochastic channels (see Figure 1B). By changing the relative firing rate of the common source with respect to the independent channels we control the correlation level λ. We set two different protocols represented in Figure 2A. In the first protocol, the correlation level is increased instantaneously from λ = 0 to some value λ > 0 at 500 ms. Therefore given that the stimulus activates the persistent state, this protocol allows us to test the effects of the correlations on the probability that the active state is erased and we refer to it as the erasing protocol. In the second protocol the correlation level is set λ > 0 for all the time, before the transient stimulus appears. In this way it is possible to see the effect of correlations on blocking the ability of the stimulus, presented during 50–100 ms, to initiate the persistent state and we refer to it as the blocking protocol.

We first demonstrate the prevalent effect of correlations: control of active memory state and control of access to the memory. In an example of the erasing protocol the excitatory stimulus activates the network into a persistent state; at 500 ms correlations are increased and the persistent state is disrupted (Figure 2A). In an example of the blocking protocol the excitatory stimulus is not able to activate the persistent state (see Figure 2B). In order to understand how these effects depend on the activity parameters we ran a large number of simulations where we injected background activity with different correlation levels for 0 ≤ λ ≤ 1 to networks with different connection probability *c* to measure how this effect spread thanks to the network architecture (Figure 2C). We compared networks with the same scaled synaptic strength *J* such that *cJN* = const. = 5.2. In the erasing protocol we estimated the erasing probability *P*_*e*_(*c*, λ) defined as the probability for the network to have the firing rate ν < 5 Hz in the interval 800–900 ms. We discarded trials where the network is not in a persistent state (ν > 5 Hz during 400–500 ms). In the blocking protocol we estimated the probability that the correlations block the stimulus; the blocking probability *P*_*b*_(*c*, λ) defined as the probability for the network to have the firing rate ν < 5 Hz in the interval 400–500 ms. This could also be seen as a gating of the persistent activity. We observe that for both protocols, the increase of both *c* and λ disrupts the persistent state: in the first case by erasing it and in the second case by blocking its activation.

We hence wanted to assess how the network size influences the stability of the persistent state under the various background activity regimes (Figure 2D). We compared networks with different size *N* with an equal average synaptic input *cJN* = const. = 5.2. We measured both erasing probability *P*_*e*_(*N*, λ) and blocking probability *P*_*b*_(*N*, λ) as a function of λ. We observe that both the erasing probability and the blocking probability (*P*_*e*_ and *P*_*b*_) increase with the network size *N*. We observe that both for fixed *c* and for fixed *N P*_*b*_ > *P*_*e*_. Finally we studied the probabilities *P*_*e*_ and *P*_*b*_ as function of *N*, fixing both *J* = 0.26 and the number of inputs that each neuron receives, i.e., *cN* = cost = 20. We computed these probabilities averaging over 500 trials. We found that with such a scaling both *P*_*e*_ and *P*_*b*_ are approximately constant (Figure 2E).

**Figure 3:**
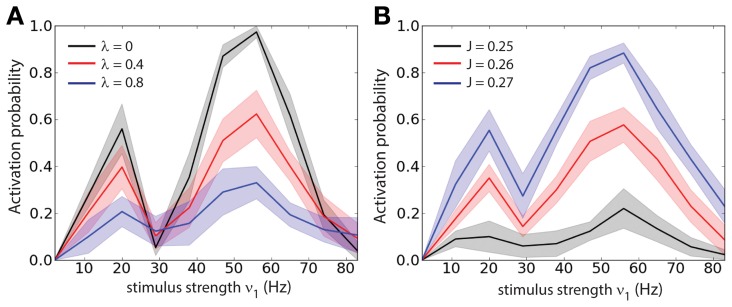
**Stimulus strength tuning is independent of the correlation level. (A)** Probability of activation (1 − *P*_*b*_) as function of the stimulus strength (ν_1_) for different values of the correlation level (λ). **(B)** Probability of activation as function of the stimulus strength (ν_1_) for different values of the recurrent synaptic strength (*J*).

In order to determine whether the optimal stimulus parameters to load of a memory (or activation of a persistent state) depend on the correlation strength we measured the loading probability (1 − *P*_*b*_) as function of the stimulus strength (ν_1_) and for different values of λ (Figure 3A), we computed the probabilities of Figure 3 averaging over 300 trials. Different values of λ change the amplitude of (1 − *P*_*b*_) but do not shift the tuning with respect to ν_1_. We also found that there are two peaks of (1 − *P*_*b*_): one at about ν_1_ ≈ 20 Hz and another at about ν_1_ ≈ 50 Hz. To test whether the positions of the two peaks depend on the recurrent network properties we measured the loading probability as function of ν_1_ and for different values of the recurrent synaptic strength *J* (Figure 3B). Similarly to the previous results, different values of *J* change the amplitude of (1 − *P*_*b*_) but do not shift the peaks of the curves with respect to ν_1_. In summary this indicates that indeed the strength of the stimulus required to active the persistent state with a set probability is dependent on the background correlations, and yet the tuning is rather broad.

**Figure 4:**
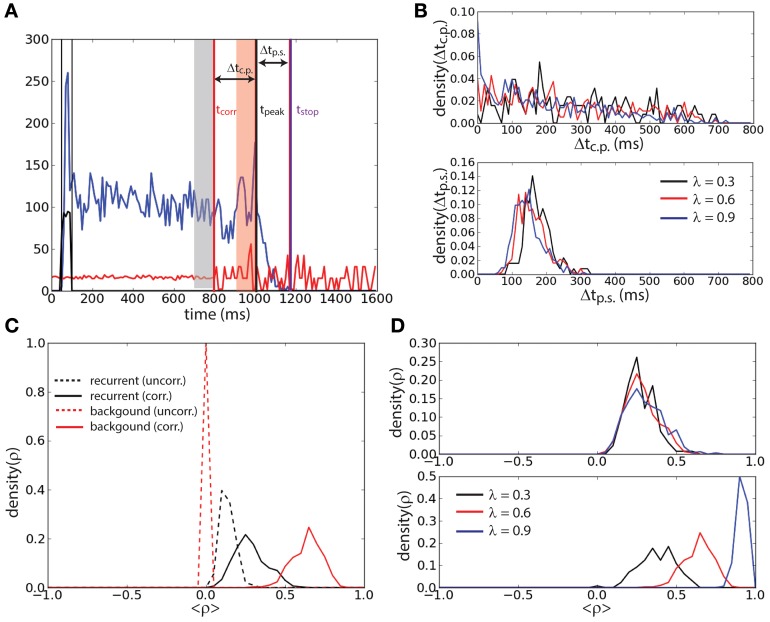
**Persistent state suppression is preceded by an increase of recurrent correlation. (A)** Timing outline of erasing persistent activity. Mean recurrent input (blue trace), mean background input (red trace), and external input (black trace) are represented together with the time at which activity is erased (*t*_stop_, magenta vertical line), time of the last peak of activity before erasing (*t*_peak_, black vertical line), and time of the onset of the correlations in background activity (*t*_corr_, red vertical line). The interval of uncorrelated activity and the interval of correlated background activity during which the correlation coefficient is measured in panels **(C)** and **(D)** are represented with gray and red shaded areas, respectively. **(B)** Distribution of the interval Δ*t*_c.p._ = *t*_peak_ − *t*_corr_ (top) and distribution of the interval Δ*t*_p.s._ = *t*_stop_ − *t*_peak_ (bottom) for different values of λ. **(C)** Mean Pearson correlation coefficient (ρ) of the recurrent input (black traces) and of the background input (red traces), computed during the uncorrelated interval (dashed traces) and the correlated interval (continuous traces). **(D)** Coefficient ρ of the recurrent input computed during the correlated interval for different values of λ (top). Same analysis for the background input (bottom).

To further investigate the effect of correlation on the stability of the persistent state we determined the lifetime of the sustained activity and the level of correlations prior to the erasing time. We defined the end of the persistent state *t*_stop_ (magenta vertical line, Figure 4A) as the first period of 10 ms (after the correlation onset) during which the firing rate of the network falls below 5 Hz. Noticing that in most of the trails a peak of activity was preceding the persistent state erasing, we defined the time of such a peak *t*_peak_ (black vertical line, Figure 4A) as the last period of 10 ms before *t*_stop_ that the firing rate attains a local maximum (in time) and that is beyond 20 Hz. We determined for each trial where the persistent state was not erased before the onset of correlations *t*_corr_ = 800 ms (red vertical line, Figure 4A), the interval Δ*t*_c.p._ = *t*_peak_ − *t*_corr_. We determined the interval Δ*t*_p.s._ = *t*_stop_ − *t*_peak_. We performed this protocol for three different values of the correlation level: λ = {0.3, 0.6, 0.9} (Figure 4B). We found that the distribution of Δ*t*_c.p._ decreases with time for all the values of correlations. When the level of correlations is larger (Figure 4B, top) the probability of reaching the peak earlier in time slightly increases with λ. Furthermore we found that the interval between the peak of activity and the erasing of the activity in the network is narrowly distributed in time. Finally this interval is independent of the correlation level, meaning that the correlations do not have a strong effect on this timing (Figure 4B, bottom). We computed these distributions averaging over 500 trials.

The mean Pearson correlation coefficient ρ (see Materials and Methods) of the synaptic input in the network during the interval with uncorrelated background activity was compared with that during the interval with correlated background activity just preceding the peak. Only trials where *t*_peak_ − *t*_corr_ > 100 ms were considered. The interval with uncorrelated background activity is defined as the 100 ms preceding *t*_corr_ (gray shaded area, Figure 4A). The interval with correlated background activity is defined as the 100 ms preceding *t*_peak_ (red shaded area, Figure 4A). We computed ρ for the background input (red lines, Figure 4C) and for the recurrent input (black lines, Figure 4C) both for the uncorrelated interval (dashed lines) and for the correlated input (continuous lines) when λ = 0.6. We found that ρ during the correlated input is smaller in the recurrent input with respect than in the background input. However, ρ of the recurrent input is larger during the correlated interval than during the uncorrelated interval. Interestingly we found that during the correlated interval, while the correlation coefficient of the background input increases with λ (Figure 4D, bottom), the correlation coefficient of the recurrent input instead remains approximately equally distributed when λ is changed (Figure 4D, top). This suggests that the network has reached the maximal amount of sustainable correlations before turning off.

**Figure 5:**
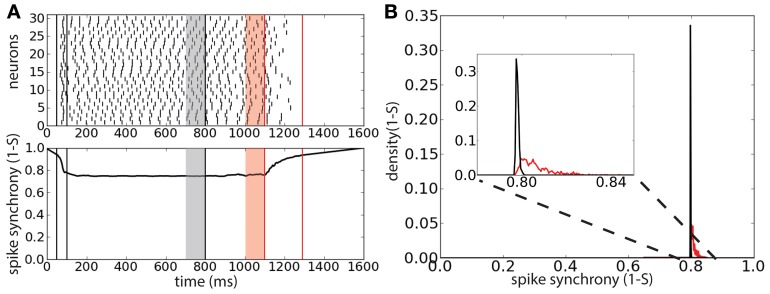
**Persistent state suppression is preceded by a weak increase of spike synchronization. (A)** Raster plot of 30 representative neurons of the network (top) and spike synchrony 1 − *S* (bottom). Same protocol of that described in Figure 4. Gray shaded area corresponds to the 100 ms interval preceding the background correlation onset (λ = 0.6); red shaded area corresponds to the 100 ms preceding the last peak of activity before sustained activity suppression. **(B)** Distribution of the average spike synchrony (1 − *S*) during the two interval described previously. Inset corresponds to a magnification of the relevant interval of spike synchrony values.

To understand whether the persistent activity deactivation is caused by an increase of spike synchrony we tracked the synchrony of the spike times using the multivariate SPIKE-distance measure *S* (Kreuz et al., 2013) (Figure 5A). The spike synchrony is given by 1 − *S* spanning the values between 0 (no synchrony) and 1 (perfect synchrony). We compared the average spike synchrony during two intervals, similarly to Figure 4: the first interval corresponds to the 100 ms preceding the start of correlated background activity and the second interval corresponds to the 100 ms preceding the last peak of activity before the deactivation of the persistent activity (provided that the onset of this last interval does not precede the start of the correlated background activity). The distribution of the average value of 1 − *S* (computed over 2000 trials) during these two intervals shows that there is a weak increase of spike synchrony preceding the persistent activity deactivation with respect to the case of uncorrelated background activity (Figure 5B). This weak increase could indicate that few spike coincidences might be the cause of the persistent activity turning off.

### Effects of background activity correlations in a winner-take-all network

We show here that modulating appropriately in space and time the correlation level of the background activity in a network performing a WM task significantly improves correct execution of all the required operations: *load, protect*, and *clear*.

We compared two different versions of the winner-take-all network; each made of two excitatory populations *B* and *R*, representing respectively colors blue and red. The two populations interact via a third population of inhibitory neurons that creates a winner-take-all mechanism. The two versions differ in that the first one receives only uncorrelated background activity while in the second each excitatory population receives also background activity from a different common noise source (Figure 1D).

We fixed the stimuli sequence as follows: during 50–150 ms a sample blue stimulus excites population *B*; during 450–550 ms a distractor red stimulus excites population *R*; during 850–950 a match blue stimulus excites again population *B*. The operations that the network has to do are to load the blue item in memory, to protect the memory at red item presentation, and to clear the memory after the match presentation.

**Figure 6:**
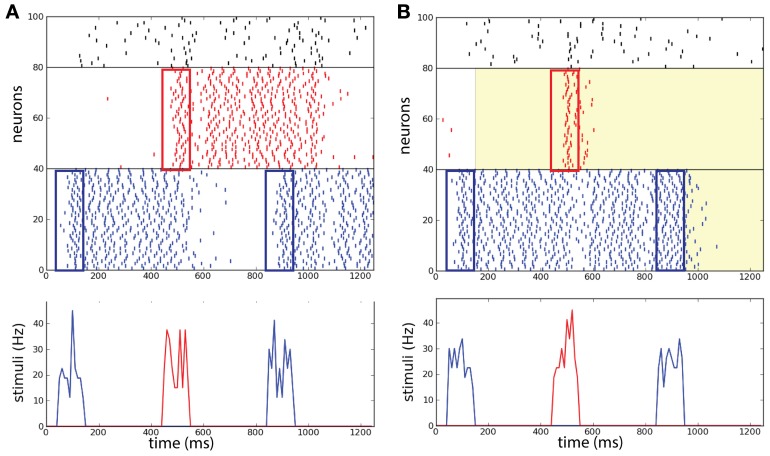
**Selective correlations implemented in a working memory task**. Two competing populations network, with two item-selective excitatory populations (blue and red) and one inhibitory non-selective population (black). **(A)** Without background correlations, the distracting stimulus activates population *R* and population *B* is deactivated. **(B)** After the activation of *B* at 150 ms, a common source of noise increases the correlations in background activity (λ = 0.9) in *R*. The correlations block the activation of *R* and maintain the persistent state *B*. After the completion of the task at 950 ms the correlations erase persistent activity in *B*. (Top) Raster plot of the neural activity in the task. (Bottom) Successively: sample stimulus to *B* (50–150 ms), distracting stimulus to *R* (450–550 ms), and match stimulus to *B* (850–950 ms).

Brunel and Wang (2001) pointed out that in order to perform the DMS task correctly, the distractor stimulus strength needs to be controlled with care: above a certain strength persistent memory-trace is perturbed by the distractor. For our case we suppose that it is reasonable to assume that all sensory stimuli in the task are of the same strength. As a preliminary test we want to confirm that in absence of background correlations the network without common noise source does not perform efficiently when the stimuli are too strong, as was already stated in the reference network described by Brunel and Wang (2001). In the example shown in Figure 6A the distractor activates *R* and via the inhibitory population the persistent state in *B* is deactivated leading to a failure of the operation *protect*.

We then consider the network represented in Figure 1D that allows the modulation of the correlation level λ in each excitatory population independently. The network initially receives uncorrelated background activity to λ = 0.9. After the first item has been loaded, the system increases the correlation level in the background activity of the other non-activated population *R*. After the match stimulus has been presented, the correlation level is increased also in population *B* to λ = 0.9.

We show an example of the network executing the WM task where the correlation level is modulated independently in the excitatory populations (Figure 6B). In this particular example we illustrate a trial where the network performs the required operations of the WM task (compare with Figure 1D and see below for statistics across trials). The distractor excites *R* only transiently such that excitation does not last enough to disrupt the activity in *B*, in addition as shown below this happens also for strong distractor stimuli. Therefore the operation *protect* has been executed with success and the memory is maintained. At the end of the match stimulus the persistent activity is disrupted also in population *B* caused by the increase of λ in that population too. Therefore the operation *clear* is executed with success and the memory is erased in the network. This example illustrates that the success of the operations *protect* and *clear* in the network with correlations are not due to the presence of inhibition as was set in the model of Brunel and Wang (2001).

**Figure 7:**
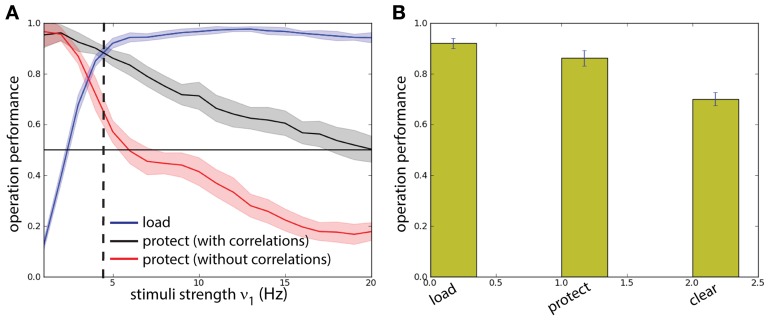
**Background correlations increase working memory performance**. Protocol reported in Figure 6. **(A)** Comparison between a two competing populations network with and without correlations. Dashed line: optimal value for the network with correlations. **(B)** The performance of the network is measured on four different operations for the optimal value of ν_1_ = 4.8 Hz: probability of activating *B* by the sample stimulus (load), probability of preventing memory disruption by a distracting stimulus in the protocol with correlations (protect), probability of erasing of the memory at the end of the task (clear). All probabilities have a high value showing that the network has good task performance, above chance.

To get quantitative measures of performance for these two networks (with and without correlations in background activity), we analyzed the statistics of *load* and *protect* performance, as a function of the stimulus intensity ν_1_ (and thus its strength) (Figure 7A). We consistently find higher *protect* performance for correlated background activity than for uncorrelated background activity throughout the whole range for ν_1_. In fact the success of the *protect* operation depends only gradually on the distractor strength. On the other hand in order to perform operation *protect* above chance level in the network with uncorrelated background activity distractors should be carefully adjusted to have intensity ν_1_ < 5 Hz. However, in this range the operation *load* is suboptimal. Hence the uncorrelated model fails in the task. This fact illustrates a recurrent problem in the protect-by-inhibition paradigm: it needs fine-tuning and achieves only low performance if the stimuli are too strong. Instead, using correlations as a mechanism to protect the activity does not need precise fine-tuning as can been seen in the large range in which both *load* and *protect* are well above chance level. We found the value ν_1_ = 4.8 Hz maximizes the joint probability of executing with success *load* and *protect* (Figure 7A, vertical dashed line). We show in Figure 7B probability of success of the three operations *load, protect*, and *clear* finding that all of them score a value higher than chance level.

### Implementing working memory task by flexible correlations modulation

We now go on to show that mutual inhibition is not a required mechanism for implementing the WM task. We show here that modulating appropriately the background activity correlations in time in a network without inhibitory population allows correct execution of all the required WM operations: *load, maintain, block*, and *clear* ( Please note that since the network studied here is made of two separated excitatory populations the component *maintain* and *block* of the operation *protect* can be treated separately).

#### Network operating regimes

In order to characterize the network performance statistics during the task we need to track three probabilities. The first probability *P*_g.o._ = *P*_*e*_*P*_*b*_ corresponds to the joint probability of deactivating by correlations the network that is in the persistent state (erase) and to block activation of the network that is in the quiescent state and is excited by a stimulus. When *P*_g.o._ dominates over the other probabilities the system is in a gate-out regime, i.e., memory cannot neither be loaded nor maintained in the network. The second probability *P*_g.i._ = (1 − *P*_*e*_)(1 − *P*_*b*_) corresponds to the joint probability that, despite the correlations, the network maintains the persistent state, if previously activated, and that the stimulus activates the persistent state when the network is in the quiescent state. When *P*_g.i._ dominates, the system is in a gate-in regime, i.e., the memory can be loaded and maintained in the network. Finally the third probability *P*_s.g._ = (1 − *P*_*e*_) *P*_*b*_ corresponds to the probability of maintaining the persistent activity in the presence of correlations while blocking the activation of a persistent state with correlated background activity when the system is in a quiescent state and is excited by a stimulus. When *P*_s.g._ dominates the system is in a selective-gate regime, i.e., the memory is maintained but cannot be loaded. In a sense we want to show that correlations in the background activity can selectively switch the network from the gate-in regime at the outset of the task, to the selective-gate regime during the memory period. We do not consider the probability *P*_*e*_ (1 − *P*_*b*_).

**Figure 8:**
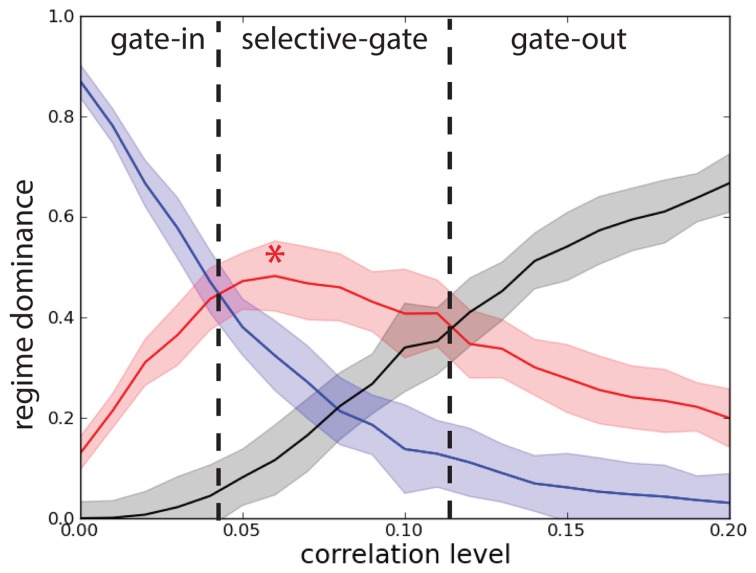
**Correlations set the network working regime**. Probability that the network operates in different regimes with network size *N* = 1000. Joint probability of (not-)erase and (not-)block: *P*_g.o._(λ) (black curve), *P*_g.i._(λ) (blue curve), and *P*_s.g._(λ) (red curve). Gate-out regime corresponds to domination of *P*_g.o._(λ) and falls in the range λ > 0.11. Gate-in regime corresponds to dominance of *P*_g.i._(λ) and falls in the range λ < 0.04. Selective-gate corresponds to dominance of *P*_s.g._(λ) and falls in the range 0.04 < λ < 0.11, with maximal value at λ = 0.07 (red star).

To obtain the network performance on the DMS task we considered the statistical results presented in Figure 2 for the single excitatory population (*N* = 1000 and *c* = 0.2). We note that there is a difference between the erasing probability *P*_*e*_(λ) and the blocking probability *P*_*b*_(λ) in function of the correlation level. In Figure 8 we present the results for network we consider in this manuscript. We see that when *P*_g.i._(λ) dominates (λ < 0.04), the system is in a gate-in regime, i.e., the memory can be loaded and maintained in the network (Figure 8). When *P*_s.g._(λ) dominates (0.04 < λ < 0.11) the system is in a selective-gate regime. We do not consider here the gate-out regime that corresponds to *P*_g.o._(λ) dominating over the other probabilities (λ > 0.11). We set the gate-in regime at λ = 0 and the selective-gate regime at λ = 0.07.

#### Modulation of correlation level in time

We now show that correlations induced by a global common noise source to the whole network executes the DMS task efficiently by modulating the correlation level λ during the different phases of the task. We note that in the mutual inhibition model, at task completion, increase in the correlation level induces the gate-out regime and erases the memory. We show here, in a two-unit model (Figure 1E), how the presentation of the match stimulus directly can erase the memory thereby implementing a direct match-based suppression without requiring inhibition. In this model each of the two excitatory populations receives background activity from sources independent to each neuron and from a noise source common to all neurons.

**Figure 9:**
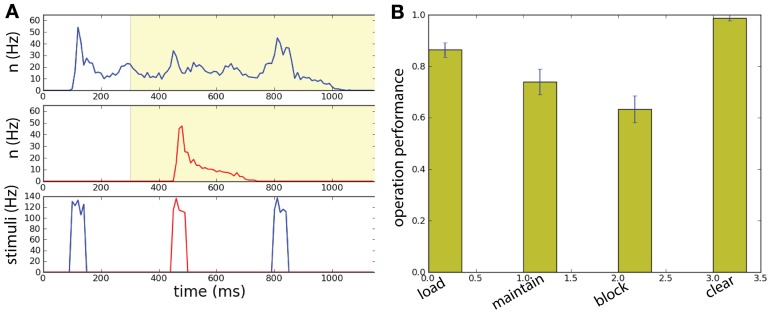
**Spatially uniform correlations in a two unit network performing a working memory task. (A)** The network has size *N* = 1000. Memory is loaded in population *B* by the sample stimulus. Background correlations block the activation of persistent population *R* by a distractor while maintaining the memory in the population *B*. The match stimulus erases the memory in population *B*, playing both the role of read-out and clear. (Top) Raster plot of the neural activity for populations *B* and *R*. (Bottom) Input current of the: sample stimulus, distracting stimulus, and match stimulus. The network has size *N* = 1000. **(B)** With intermediate correlation level (λ = 0.07) in background activity the network can execute successfully the working memory task. Task performance of the network for the four operations: load, block, maintain, clear.

An example of the network performing the DMS task is represented in Figure 9A. The stimuli are presented in the following sequence: sample stimulus to population *B* at time during 100–150 ms, distractor stimulus to population *R* during 450–500 ms, and match stimulus to population *B* at time 800–850. In the beginning the system is in the gate-in regime (λ = 0): the sample stimulus activates *B*. From 300 ms the network is set in a selective-gate regime (λ = 0.07): the distractor stimulus activates transiently population *R* while persistent activity is maintained in population *B*. At the end of the task the match stimulus, first, increases the activity in *B* and, then, destroys it.

We can then compute the task performance of the network, corresponding to the success rate that the operations *load, maintain, block*, and *clear* are executed successfully (Figure 9B). These measure are all above chance level. Notice the high level of performance of the *clear* operation.

## Discussion

### Results and data discussion

In this work we present a novel paradigm explaining how the persistent activity can be modulated on-line by the mean of both information-related signal and background activity. This paradigm is based on our result showing that background correlations influence the transition between the persistent state and the quiescent state in a bistable recurrent neural network. We call this phenomenon correlation-induced gating.

In order to implement a multi-unit network performing a WM task, we began by establishing the basis of the correlation-induced gating on a single-unit network. We show that background correlations block and erase a persistent state in a homogeneous recurrent neural network representing a single unit. We found that the transition rate from the persistent state to the quiescent state increases, with the network size and with the connection probability. For all situations the probabilities increase with the correlation level. Increasing the network size, while fixing the connections probability and renormalizing the synaptic inputs to keep the average input strength constant scales up the probabilities. In other words, in larger size network with weaker but more numerous synapses, correlations appear to have a stronger effect. On the other hand, when we fix the total number of synapses each neurons receives, growing network size does not appear to have much effect on the correlation driven probabilities. These effects could be related to the fact that the amount of correlation between neurons sharing common input is mainly determined by pooling (Rosenbaum et al., 2010, 2011).

We implemented a winner-take-all network composed of two excitatory populations and one inhibitory population. Each of the excitatory populations receives background input from independent noise sources and a noise source common to the neurons of such population. The amount of correlation could be changed independently in the two excitatory populations. By increasing the level of correlations in the populations encoding an irrelevant information we prevented a distractor from loading a memory item in such population. In particular we showed that this model allows to prevent stronger distractors with respect to a model inspired by Brunel and Wang (2001) where the distractor is blocked only by the mutual inhibition. Our model could therefore explain how the response to the distractor stimulus in a WM task could be as strong as for the sample stimulus (Miller et al., 1996). This effect would be in fact not compatible with a model where a distractor is prevented by mutual inhibition.

We implemented a WM network differing by the same previous one in the construction of background correlations that are induced by a shared source. We show that modulating the correlation level in background activity we can set the system in the different regimes. This time instead of modulating the correlations level “in space” we modulate it in time. Depending on the strength of the correlations the system is set in different operating points, namely the gate-in, selective-gate, and gate-out regimes. The gate-in regime allows to load a memory in the WM store and to maintain it subsequently. The selective-gate regime maintains a previously loaded memory but blocks the load of any new memory. The gate-out regime blocks the network both to load and to maintain a memory. We can switch instantaneously from one dynamic regime to the other by tuning the strength of the background activity correlations. We further show that the projection of a strong match stimulus can be sufficient to clear the memory at task completion, thereby suggesting that correlations also play a role in match-suppression.

We must also note that in this work we considered spatial correlations and their effect on the persistent activity and WM task executions. In a companion paper we have shown that temporal structure also has an important effect: the gating modes are modulated by the oscillatory frequency content of the background activity (Dipoppa and Gutkin, 2013). While in the companion paper the block and erase probabilities, and thus the gating modes of the network, are modulated by the oscillation frequency, in this work they are modulated by the correlation level. As opposed to the non-monotone relationship between the oscillation frequency and the block and erase probabilities [Figure 3A of Dipoppa and Gutkin (2013)], there is a monotone relationship between the correlation level and the same measures (Figures 2C,D). Hence control of the WM through spatial correlations could be implemented by a simple increase or decrease of activity within a neural population furnishing connections common to the WM store, while the oscillatory control would require more complex task-dependent shifting between the frequency bands. We would like to speculate that the two mechanisms could represent two independent modes of control over the WM networks. Furthermore, in this work we uniquely examine the role of mutual inhibition and show that the spatial correlation structure alleviates the network sensitivity to stimulus strength.

Although we do not propose a mechanism for the read-out of the memory information we note that the mechanism proposed by Brunel and Wang (2001) for their network would be compatible with our model. This mechanism corresponds to the fact that a match stimulus will elicit a stronger response with respect to a distractor stimulus in the first few tenths of milliseconds since the first will excite a network that is already in the persistent state. Hence we might speculate that a complementary population of neurons sensitive to rapid transients in the activity might be a way to signal read-out differentially.

### Model predictions and open questions

The novel paradigm that we present here allows to manipulate persistent activity through background correlations. An advantage of the correlation-induced gating with respect to the inhibition-induced gating is that the gate can be rapidly and flexibly opened or closed depending on the correlation level, instead of being fixed by the network connectivity structure.

The effects that we find for the flexible changes in the correlation levels is the major prediction of the model. We predict that an examination of multi-unit electrophysiological recordings of animals performing a WM task will show the following modulation of correlation level: low level during loading and intermediate level during maintenance (as in the two-unit model of Figure 1E) or alternatively high level of correlations in the population of neuron selective for a non-memorized item during the delay period (as in the winner-take-all model of Figure 1D). To our knowledge experiments specifically analyzing how correlations change in the PFC as the delay-response task unfolds are still lacking.

At the same time, there are several lines of indirect evidence that lead us to believe that task dependent correlation modulation is indeed possible. First, it has been found that there is a modulation of spike coincidences during different phases of a motor task (Riehle et al., 1997). Riehle et al. (1997) found that at times during the delay when the animal was expecting to generate a response there were transients of synchronized spikes. Furthermore for successful trials there were more synchronized spikes during the delay period than for failure trails. This indeed suggests that spike coincidence is modulated in a functional way. The increase of excess synchrony at response (or expected response) times is compatible with the correlation based memory clearance discussed in this manuscript. Furthermore, it has been found that a change in representation during the delay-response task leads to an increase of synchronization (Sakamoto et al., 2008). Pipa and Munk (2011) analyzed multi-unit activity during the delay period of a match-to-sample task and found that on correct vs. incorrect trials there is a modulation of spike synchronization and further, synchronous spike events are more prevalent at match presentation. This last point again suggests that increased correlations may be involved in erasing the memory trace.

In fact there is ample literature relating changes in oscillatory synchrony, coherence and frequency during WM tasks (Tallon-Baudry et al., 1998; Pesaran et al., 2002; Lee et al., 2005; Pipa et al., 2009). For example Pesaran et al. (2002) found that gamma-band spiking coherence is increased during the delay period in the lateral intraparietal cortex (LIP) in primates performing a delayed response task. Given that LIP is coupled to the PFC and is also involved in WM trace (Chafee and Goldman-Rakic, 1998), this is suggesting of increased input correlations to the PFC during the WM task. In the context of irregular poisson firing, oscillatory coherence is nothing other than correlations organized both in time (the frequency) and space. Oscillatory effects are beyond the scope of this paper and are a subject of the companion manuscript (Dipoppa and Gutkin, 2013).

The data reviewed above does show that there is a modulation of activity correlations during the WM task, yet it does not provide the mechanism. Here we propose that the mechanism is in the background input correlations generated by a common source. One hence might ask where such inputs may be coming from. As hinted above, one source could be coherent firing activity in the cortical regions coupled to the PFC and involved in WM processing (e.g., LIP). In addition, we propose that the source of shared background input generating spatial correlations can reside in the striatum, a subcortical area thought to be involved in WM. In fact the structure of the cortico-striatal loops as been longly seen as a disadvantage for the WM capacity if the memory store is located also in the striatum. Since the number of striatal neurons is much lower than the number of pyramidal neurons (Lange et al., 1976) and the loop is based on divergence (resp. convergence) in the striato-cortical (resp. cortico-striatal) direction, then the striatum could not have the same memory capacity of the cortex. It has been suggested that instead that divergent/convergent structure could be useful since the basal ganglia do not encode the individual information of WM but they control the gate of other region and decide when they can be updated (Frank et al., 2001). We also suggest that striatum plays a gating role since it could be the source of the common noise that creates the different regimes.

The correlation-induced gating is a robust effect to parameters variation. We propose the following explanation for this phenomenon: background correlations induce spike-times synchronization in the recurrent network, as was found similarly for independent neurons by Galán et al. (2006), and this leads to persistent activity erasing and block because of the refractory period of the neurons, as was found by Laing and Chow (2001) and Gutkin et al. (2001). Providing a proof of this assumption and a mathematical explanation of the correlation-induced gating will be the subject of future research.

### Conflict of interest statement

The authors declare that the research was conducted in the absence of any commercial or financial relationships that could be construed as a potential conflict of interest.
